# MicroRNA let‐7g possesses a therapeutic potential for peripheral artery disease

**DOI:** 10.1111/jcmm.12997

**Published:** 2016-10-03

**Authors:** Po‐Yuan Hsu, Edward Hsi, Tzu‐Ming Wang, Ruey‐Tay Lin, Yi‐Chu Liao, Suh‐Hang H. Juo

**Affiliations:** ^1^ Department of Medical ResearchChina Medical University HospitalTaichungTaiwan; ^2^ Department of NeurologyKaohsiung Medical UniversityKaohsiungTaiwan; ^3^ Department of NeurologyNational Yang‐Ming University School of MedicineTaipeiTaiwan; ^4^ Department of NeurologyTaipei Veterans General HospitalTaipeiTaiwan; ^5^ Graduate Institute of Biomedical SciencesChina Medical UniversityTaichungTaiwan

**Keywords:** microRNA let‐7g, vascular endothelial growth factor‐A, endothelial progenitor cells, peripheral artery disease, angiogenesis

## Abstract

Peripheral artery disease (PAD) is a manifestation of systemic atherosclerosis and conveys a significant health burden globally. Critical limb ischaemia encompasses the most severe consequence of PAD. Our previous studies indicate that microRNA let‐7g prevents atherosclerosis and improves endothelial functions. This study aimed to investigate whether and how let‐7g therapy may improve blood flow to ischaemic limbs. The present study shows that let‐7g has multiple pro‐angiogenic effects on mouse ischaemic limb model and could be a potential therapeutic agent for PAD. Mice receiving intramuscular injection of let‐7g had more neovascularization, better local perfusion and increased recruitment of endothelial progenitor cells after hindlimb ischaemia. The therapeutic effects of let‐7g's on angiogenesis are mediated by multiple regulatory machinery. First, let‐7g increased expression of vascular endothelial growth factor‐A (VEGF‐A) and VEGF receptor‐2 (VEGFR‐2) through targeting their upstream regulators HIF‐3α and TP53. In addition, let‐7g affected the splicing factor SC35 which subsequently enhanced the alternative splicing of VEGF‐A from the anti‐angiogenic isoform VEGF‐A_165b_ towards the pro‐angiogenic isoform VEGF‐A_164a_. The pleiotropic effects of let‐7g on angiogenesis imply that let‐7g may possess a therapeutic potential in ischaemic diseases.

## Introduction

Peripheral artery disease (PAD) is often associated with diabetes and coronary artery disease, leading to significant morbidity and mortality 1, 2. The prevalence of asymptomatic PAD is estimated to be around 3–10%, increasing to 15–20% in persons over 70 years 3. Critical limb ischaemia (CLI) is the most severe clinical manifestation of PAD, which causes intermittent claudication, gangrene and foot ulceration. Patients with CLI are at risk of devastating complications including amputation and mortality 4, 5.

MicroRNAs (miRNAs) are small (~22 nucleotide long) non‐coding RNAs that regulate gene expression at the post‐transcriptional level by degradation of mRNAs or inhibition of protein translation 6. The miRNA let‐7 family plays a pivotal role in cell proliferation, cancer and cardiovascular diseases 7, 8. Our group previously reported that let‐7g prevents atherosclerosis by inhibiting the uptake of oxidized‐low density lipoprotein (ox‐LDL) into endothelial cells (ECs) and vascular smooth muscle cells 9. Our recent study further showed that reduced let‐7g levels impairs endothelial functions through targeting transforming growth factor beta (TGF‐β) and sirtuin‐1 (SIRT‐1) signalling pathways 10. We demonstrated that low let‐7g levels can increase thrombosis, inflammation, senescence and anti‐angiogenesis, all of which contribute to vascular diseases. Another group showed that hypoxia could up‐regulate the let‐7 family (including let‐7g) in ECs, which in turns targeted argonaute 1 (AGO1) leading to de‐suppression of vascular endothelial growth factor‐A (VEGF‐A) 11. Another recent study showed that PAD patients had elevated levels of anti‐angiogenic VEGF‐A isoform (VEGF‐A_165b_) and decreased levels of pro‐angiogenic isoform VEGF‐A_165a_ (the human orthologue of murine VEGF‐A_164a_) 12. Therefore, an increase in let‐7g may be beneficial to PAD patients.

This study aimed to investigate whether let‐7g therapy could improve blood flow to ischaemic tissue in the hindlimb ischaemic animal model. The effects of hypoxia on the let‐7g and let‐7g‐regulated genes were investigated in muscle cells. Let‐7g's effects on VEGF‐A, vascular endothelial growth factor receptor 2 (VEGFR‐2), endothelial progenitor cells (EPCs) and the underlying mechanisms were explored.

## Materials and methods

### Muscle cell culture

C2C12, a mouse myoblast cell line, was used for the *in vitro* studies. C2C12 cells were grown in high glucose Dulbecco's modified Eagle's medium (DMEM; Thermo Scientific, Waltham, MA, USA) and supplemented with 10% foetal bovine serum (FBS). For the hypoxic experiment, the C2C12 cells were cultured under 1% O_2_, 5% CO_2_ and 94% N_2_ for 24 hrs. For the oxygen glucose deprivation (OGD) experiment, the C2C12 cells were cultured using an identical hypoxia protocol, but the cells were incubated in DMEM medium without glucose and serum (Thermo Scientific) at 37°C for 6 hrs. Following the OGD insult, fresh culture medium was added in the cultures. The cells were then allowed to re‐oxygenate under normal conditions (37°C, 5% CO_2_, 95% air) for 18 hrs. For the siRNA study, C2C12 cells were incubated at 37°C in a humidified atmosphere for 24 hrs. C2C12 cells were transfected with different doses of CDK6 siRNA (5, 10, 50, 100 nM) or control siRNA for 24 hrs.

### Animal model of hindlimb ischaemia

Ten‐week‐old male C57BL/6 mice (Charles River Technology; BioLASCO Taiwan Co., Ltd, Taipei, Taiwan), weighing 22–25 g were used for all experiments. To produce hindlimb ischaemia, mice were sedated with isoflurane (Abbott Laboratories Ltd., Queenborough, Kent, United Kingdom), and anaesthetized by intraperitoneal administration of pentobarbital sodium (40 mg/kg; Sigma‐Aldrich, St. Louis, MO, USA). The mice were placed in a supine position on a warming pad at 37°C with the left hindlimbs shaved. The left femoral artery and all attached side‐branches were dissected free and then excised along its entire length. The veins were left intact during the procedure and the overlying skin was then closed 13.

The negative control‐miRNA and let‐7g mimic were from Thermo Scientific. Both types of miRNAs were formulated with a commercial PEI‐based nanoparticle (called *in vivo*‐jetPEI^®^) for animal studies. Mice then were divided into two groups (*n* = 6 for each group) for intramuscular injection (IM) of 5 nM negative control‐miRNA (placebo group) or let‐7g mimic (let‐7g group). IM injection was given at three sites of the gastrocnemius muscle at the medial thigh on day 1, 8 and 15 after induced hindlimb ischaemia 14. Noticeably, the day for induction of ischaemic limb was defined as day 0. All animal procedures were compliant with the standards and approved protocols by Kaohsiung medical university‐institutional Animal Care and Use Committee (IACUC).

### Measurement of hindlimb perfusion

The blood flow of ischaemic (left) limb and normal (right) limb was measured using the Laser Doppler Perfusion Image (LDPI, Moor‐LDI2‐2λ; Moor, Co., United Kingdom) on day 2, 7, 14 and 21 postoperatively. The LDPI system uses a near infrared laser beam (633 and 830 nm) and has an average measurement depth of approximately 1.0–2.0 mm. This imaging technique provides a non‐invasive measurement of blood flow by determining the Doppler frequency shift of light reflected by the moving red blood cells 15. During the scanning procedure, mice were placed in a supine position on a warming pad at 37°C and the normal and ischaemic regions were identified. The machine then focused on each region and consecutively measured the intensity of blood flow over the region of interest (leg and foot). Colour‐coded images were recorded, and analyses were performed automatically by calculating the average perfusion of each leg (ischaemic and non‐ischaemic legs separately). The perfusion ratio calculated as blood flow in left (ischaemic) *versus* right (normal) legs of each animal was used to assess the circulation to the ischaemic limb.

### Immunohistochemistry to measure capillary density

Capillary density, as an index of angiogenesis, was determined by counting the total number of endothelial cells relative to the total surface area *via* light microscopy. Ischaemic (left) leg of each animal was harvested on day 21 after surgery and embedded in 10% formalin. The gastrocnemius muscle was excised and fixed overnight in 10% formalin in phosphate‐buffered saline (PBS). Paraffin‐embedded sections of 5 μm‐thickness muscle sections were first treated with 0.3% H_2_O_2_ for 30 min. and incubated with block reagent for 1 hr at room temperature. Sections were then incubated with the primary antibody specifically against CD31 (1:100; GeneTex, Irvine, CA, USA) for 1 hr, followed by incubation with biotinylated secondary antibody (1:200). The capillary density was defined as: (capillary surface area)/(total surface area of each section) (×100%). The capillary density for each mouse was counted under five randomly selected 200× fields using Image J software.

### Definition of EPCs

Bone marrow and peripheral blood were obtained from each mouse on day 21 after induction of hindlimb ischaemia. To obtain whole marrow cells, femoral and tibial bones were aseptically harvested and the bone marrow cavity was washed with DMEM. Marrow cells were then centrifuged at 300 × *g* for 10 min. and the supernatant was removed. Mononuclear cells were isolated from peripheral blood by gradient centrifugation using Histopaque 1083 (Sigma‐Aldrich). To determine the proportion of EPCs in bone marrow and peripheral blood, whole marrow cells and circulating mononuclear cells were incubated with monoclonal antibodies against CD34 and VEGFR‐2 for 1 hr. Secondary detection was performed using Alexa Fluor 488‐ and Texas Red 615‐conjugated secondary antibodies (BioSmart, Houston, TX, USA). Isotype‐identical antibodies (IgG) were served as controls. Flow cytometry analyses were performed by utilizing a fluorescence‐activated cell sorter (FACS, Beckman Coulter FC500 flow cytometer). Cells carried both CD34 and VEGFR‐2 (CD34^+^/VEGFR‐2^+^) were defined as EPCs.

### mRNA and protein analysis

The gastrocnemius muscles of ischaemic and non‐ischaemic limbs were harvested on day 21 after mice were killed. Total RNA and protein extracted from the gastrocnemius muscle or C2C12 cells were used to analyse the expression levels of VEGF‐A, VEGF‐A_164a_, VEGF‐A_165b_, VEGFR‐2, Hypoxia‐inducible factor 3‐alpha (HIF‐3α), Tumour Protein 53 (TP53), Cyclin D‐dependent kinases 6 (CDK6) and splicing factor SC35. Total RNA was extracted with TRIzol^*®*^ Reagent (Invitrogen), and cDNA was produced using 1 μg of starting mRNA (Applied Biosystems, Darmstadt, Germany) and random hexamers. The sequences of PCR primers are shown in Table 1, and primer sequences for VEGF‐A_164a_ and VEGF‐A_165b_ were from Kikuchi *et al*. 12. Quantitative real‐time PCR (qPCR) was performed with an ABI PRISM 7900 sequence detector and SYBR green reagents (Applied Biosystems, Waltham, MA, USA). All samples were run in triplicate. The relative amount of mRNA of interest was normalized to 18S rRNA. For let‐7g level quantification, U6 was used as the internal control. Dissociation curves were performed to confirm the specificity of PCR products.

**Table 1:** Primers used for quantitative real‐time polymerase chain reaction

Gene	Primer
VEGF‐A F	5′‐AAC GAA AGC GCA AGA AAT CC‐3′
VEGF‐A R	5′‐GCT CAC AGT GAA CGC TCC AG‐3′
VEGF‐A_164a_ F	5′‐C AGA AAA TCA CTG TGA GCC TTG TT‐3′
VEGF‐A_164a_ R	5′‐C TTG GCT TGT CAC ATC TGC AA‐3′
VEGF‐A_165b_ F	5′‐C AGA AAA TCA CTG TGA GCC TTG TT‐3′
VEGF‐A_165b_ R	5′‐C TTT CCG GTG AGA GGT CTG C‐3′
VEFGR‐2 F	5′‐CCA CCC CAG AAA TGT ACC AAA C‐3′
VEGFR‐2 R	5′‐AAA ACG CGG GTC TCT GGT T‐3′
18S F	5′‐TTG ATT AAG TCC CTG CCC TTT GT‐3′
18S R	5′‐CGA TCC GAG GGC CTC ACT A‐3′
HIF‐3 F	5′‐TGT GAA CTT CAT GTC CAG GC‐3′
HIF‐3 R	5′‐GCA ATG CCT GGT GCT TAT CT‐3′
TP53 F	5′‐CAC GTA CTC TCC TCC CCT CAA T‐3′
TP53 R	5′‐AAC TGC ACA GGG CAC GTC TT‐3′
CDK6 F	5′‐TCT CAC AGA GTA GTG CAT CGT‐3′
CDK6 R	5′‐CGA GGT AAG GGC CAT CTG AAA A‐3′
SC35 F	5′‐TCC AAG TCC AAG TCC TCC TC‐3′
SC35 R	5′‐ACT GCT CCC TCT TCT TCT GG‐3′

Muscle tissue were homogenized in RIPA buffer (150 mM NaCl, 1% NP‐40, 0.5% deoxycholic acid, 0.1% SDS, and 50 mM Tris) (GeneTex), and insoluble constituents were removed by centrifugation. Protein lysates were denatured and loaded onto a 4‐12% SDS polyacrylamide gel. The separated proteins were then transferred onto a PVDF membrane (Millipore, Billerica, MA, USA) and blocked with 5% non‐fat dry milk for 2 hrs at room temperature. The membrane was incubated overnight at 4°C in 5% non‐fat dry milk /PBST containing the primary antibodies. Primary antibodies against VEGF‐A (0.1 μg/ml; R&D, Minneapolis, MN, USA), VEGF‐A_165b_ (2 μg/ml; Millpore, Billerica, MA, USA), VEGFR‐2 (1:1000; Cell Signaling, Danvers, MA, USA), TP53 (0.25 μg/ml; R&D), Hif‐3α (1:1000; Santa Cruz Biotechnology, Santa Cruz, CA, USA), SC35 (1:2000; Abcam, Cambridge, United Kingdom), CDK6 (1:2000; Cell Signaling) and α‐tubulin (1:5000; ProteinTech Group, Cambridge, United Kingdom) were used. The membrane was incubated with the secondary antibody conjugated to horseradish peroxidase. The ECL non‐radioactive detection system was used to detect the antibody–protein complexes by LAS‐3000 imaging system (Fujifilm, Tokyo, Japan). Blot intensity was quantitatively measured by ImageJ software (NIH).

### Reporter constructs, mutagenesis and luciferase reporter assay

We used Ingenuity Pathways Analysis (IPA; https://analysis.ingenuity.com) to map molecular pathways and networks for the prediction of let‐7g targets. The IPA Database is a constantly curated resource of published literature on gene functions and interactions. For luciferase reporter assay, a PCR fragment containing the let‐7g binding site and surrounding 3′UTR sequence (300 bp) of each gene was subcloned into the pMIR‐REPORT^™^ luciferase vector (Ambion, Foster City, CA, USA). Since there were two let‐7g binding sites in HIF‐3α 3′untranslated region (3′UTR), two plasmid constructs, HIF‐3α‐1 (let‐7g binding site at position 2181 to 2188) and HIF‐3α‐2 (let‐7g binding site at position 2320 to 2326) were created. The mutant construct of each candidate genes were created by site mutation in seed sequence of let‐7g binding site using QuikChange Lightning Site‐Directed Mutagenesis Kit (Agilent Technologies, Santa Clara, CA, USA). For luciferase reporter assay, the reporter constructs were transiently transfected into HEK293 cells along with different doses of let‐7g mimic (0, 5, 10 and 25 nM) using the HiPerFect Transfection Reagent (Qiagen, Hilden, Germany). pEGFP plasmids (100 ng) were cotransfected to serve as the internal control for transfection efficacy. The luciferase activity was measured using the Luc‐Pair^™^ miR luciferase assay kit (GeneCopoeia, Rockville, MD, USA).

### Statistical analysis

Quantitative data are expressed as mean ± S.E.M. Student's *t*‐test was used to compare differences in parameters between let‐7g group and placebo group. A probability value <0.05 was considered statistically significant.

## Results

### Let‐7g increases perfusion in the rodent ischaemic hindlimb

**Figure 1:**
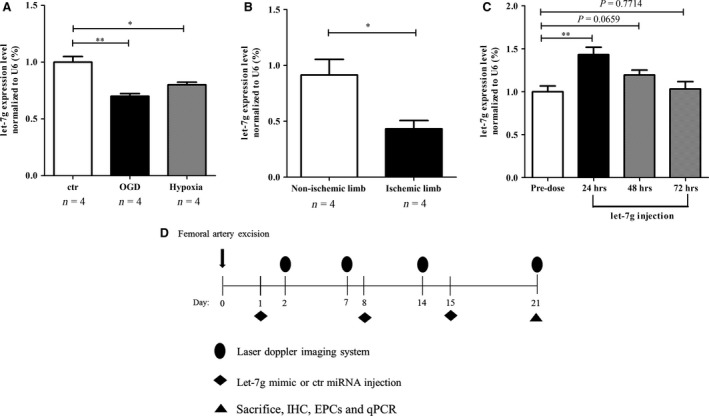
Ischaemia reduces let‐7g level. (**A**) let‐7g level was decreased in the C2C12 cells subjected to hypoxia or oxygen glucose deprivation (OGD). (**B**) let‐7g level was decreased in the ischaemic limbs. (**C**) A measurement of let‐7g delivery efficacy after let‐7g intramuscular injection to normal mice. (**D**) The process of experiment of hindlimb ischaemia. Data are presented as mean ± S.E.M., *P < 0.05, **P < 0.01. ctr: control; miRNA: microRNA; IHC: Immuno‐histochemistry; EPCs: endothelial progenitor cells; qPCR: Quantitative real‐time polymerase chain reaction.

The let‐7g level was decreased in C2C12 cells under the hypoxia or OGD condition (Fig. 1A). In addition, our *in vivo* data showed that let‐7g level was significantly decreased in the ischaemic limb (Fig. 1B). Injection of let‐7g to hindlimbs of normal mice significantly increased let‐7g levels in gastrocnemius muscles at 24 hrs, then the level declined gradually at 48 hrs, and returned to the baseline at 72 hrs (Fig. 1C). The timeframe of the protocol for animal experiment of hindlimb ischaemia is shown in Fig. 1D.

**Figure 2:**
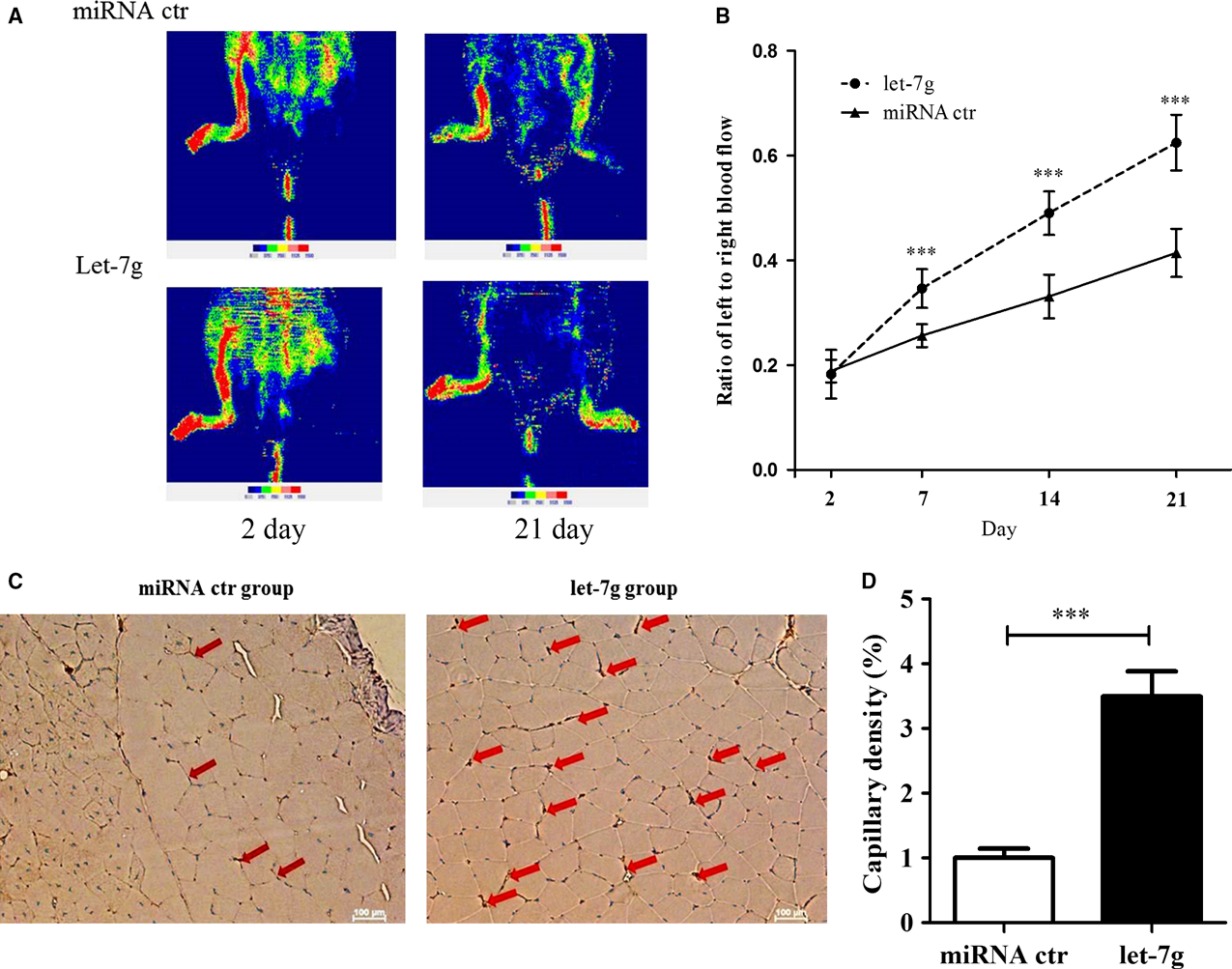
Let‐7g treatment to animals of hindlimb ischaemia. (**A**) Representative images of Laser Doppler perfusion imaging to quantify regional blood flow on day 2 and day 21. (**B**) The comparison of perfusion ratio (blood flow in the left ischaemic limb to the right normal limb) between let‐7g and miRNA control (miRNA ctr) treatment at different time‐points. (**C**) Representative photographs of CD31 immunohistochemistry staining for capillary densities in the ischaemic gastrocnemius muscle on day 21. Scale bar: 100 μm (**D**) Quantification of capillary density is calculated as capillary surface area divided by total surface area of each section (×100%). Data are presented as mean ± S.E.M.,* n* = 6; ***P < 0.001.

The operation of ligation and excision of the left femoral artery effectively reduced the perfusion ratio to 0.20 at 48 hrs post‐operation for mice in either let‐7g‐treated or placebo group (Fig. 2A and B). The perfusion ratio was significantly increased in let‐7g‐treated mice compared with placebo‐treated mice on day 7 (0.346 ± 0.015 *versus* 0.256 ± 0.009, *t*‐test *P* = 0.0004). Representative images of the hindlimb perfusion on day 2 and day 21 are shown in Fig. 2A. In the let‐7g‐treated group, the perfusion ratio improved promptly and reached to 0.63 on day 21 post‐operation (Fig. 2B).

### Capillary density analysis

To confirm that the improved perfusion ratio is attributed to neo‐vasculization, immunohistochemistry staining with anti‐CD31 antibody was performed in the gastrocnemius muscle of ischaemic legs. Representative photographs of histological sections are shown in Fig. 2C. The capillary density in the gastrocnemius muscle was significantly higher in the let‐7g‐treated group than that in the placebo group on day 21 post‐operation (3.50 ± 0.39% *versus* 1.00 ± 0.14%, *P* = 0.0001) (Fig. 2D).

### Let‐7g enhances EPC recruitment

**Figure 3:**
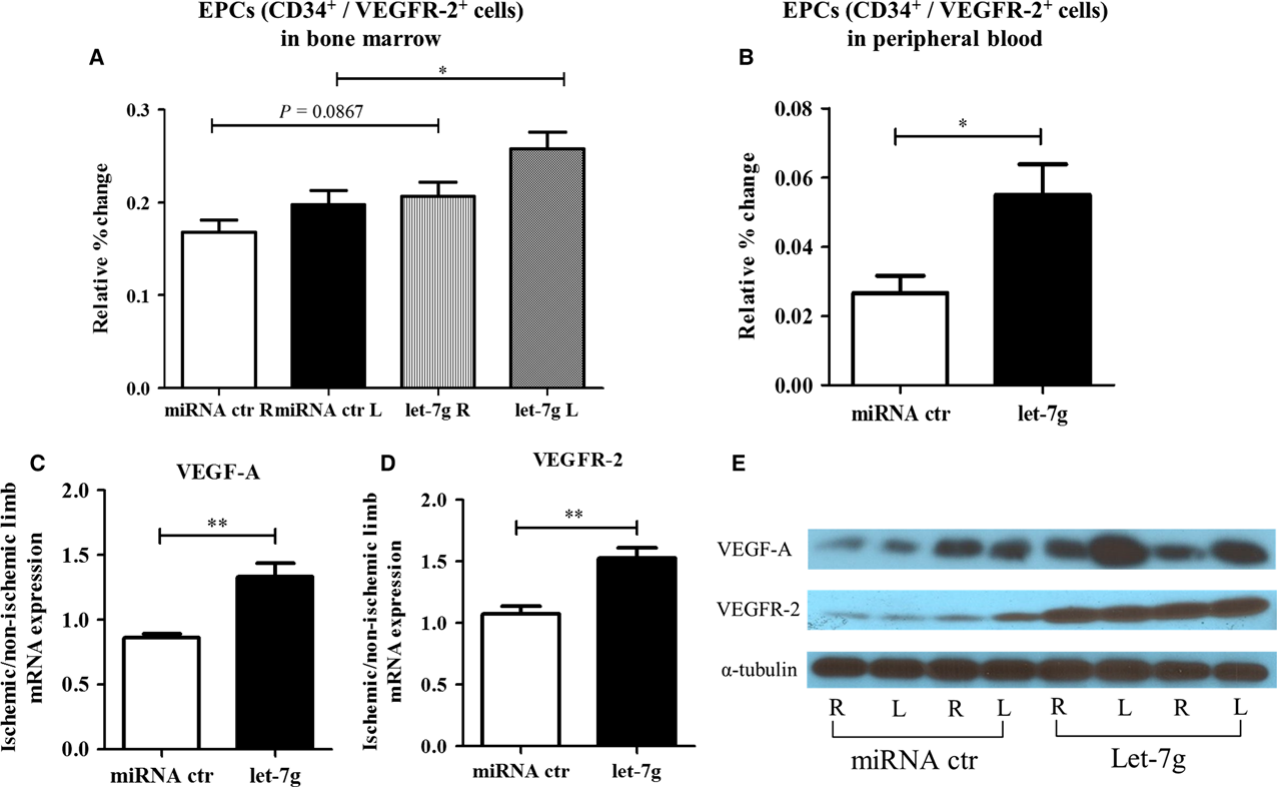
Let‐7 g enhances EPC recruitment and VEGF‐A and VEGFR‐2 expression. (**A**) The proportion of bone marrow derived EPCs (CD34^+^/ VEGFR‐2^+^ cells) level in the ischaemic (left) and non‐ischaemic (right) leg of mice on day 21 after femoral artery excision (*n* = 6). (**B**) The proportion of circulating EPCs (CD34^+^ / VEGFR‐2^+^ cells) in the peripheral blood was significantly higher in let‐7g treatment group than that in the miRNA control (miRNA ctrl) group (*n* = 6). (**C, D**) The mRNA ratio of total VEGF‐A and VEGFR‐2 in the ischaemic (left) legs versus non‐ischaemic (right) legs (*n* = 6 per group) (**E**) western blot data show that the ratios of VEGF‐A and VEGFR‐2 protein levels are higher in the let‐7 g‐treated than the miRNA control‐treated animals by 1.45× and 1.20×, respectively (*n* = 2 for each group). Data in the bar figures are presented as mean ± S.E.M., *P < 0.05, **P < 0.01.

Mobilization and recruitment of EPCs (defined as CD34^+^/VEGFR‐2^+^ cells) to the ischaemic region are important for neo‐vascularization. Flow cytometry analysis was performed to detect EPCs in the bone marrow and in peripheral mononuclear cells. On day 21, EPC percentage among the bone marrow cells was highest in the ischaemic leg of let‐7g‐treated group and lowest in the non‐ischaemic leg of placebo group (0.258 ± 0.018% *versus* 0.168 ± 0.013%, *P* = 0.0272) (Fig. 3A), while the data were similar between non‐ischaemic legs of let‐7g‐treated group (0.207 ± 0.015%) and ischaemic legs of placebo group (0.198 ± 0.015%). Additionally, the percentage of EPCs in the circulating mononuclear cells was significantly higher in the let‐7g treated group than in the placebo group (0.055 ± 0.009% *versus* 0.027 ± 0.005%, *P* = 0.019) (Fig. 3B).

### Let‐7g effects on VEGF‐A and VEGFR‐2

To assess the role of let‐7g in the VEGF‐A signal pathway, we analysed the expression ratio of VEGF‐A and VEGFR‐2 in gastrocnemius muscle of the ischaemic/non‐ischaemic hindlimb. Twenty‐one days after the induction of hindlimb ischaemia, mRNA ratios of VEGF‐A and VEGFR‐2 were significantly higher in let‐7g‐treated group than those in the placebo group (Fig. 3C and D, *P* = 0.0017 and 0.0017 for VEGF‐A and VEGFR‐2, respectively). Similarly, the ratios of VEGF‐A and VEGFR‐2 protein levels of ischaemic/non‐ischaemic hindlimb are higher in the let‐7g‐treated than placebo‐treated animals (Fig. 3E). Using the ratio from placebo‐treated animals as the reference, the relative ratios for let‐7g‐treated animals were 1.45 for VEGF‐A protein and 1.20 for VEGFR‐2 protein.

### HIF‐3^α^ and TP53 as let‐7g target genes

**Figure 4:**
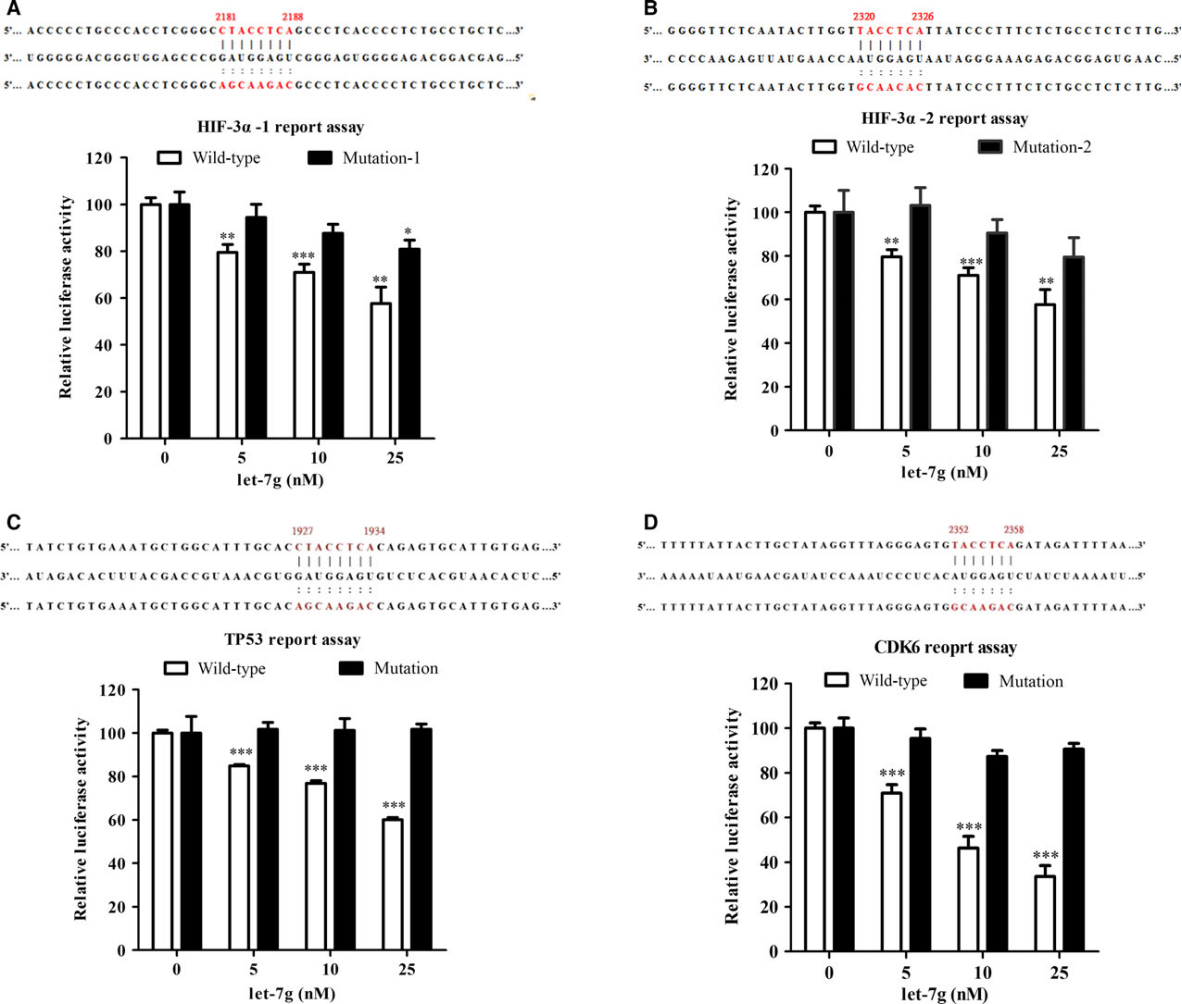
Luciferase reporter assay for let‐7g binding sites in the 3′‐UTR of HIF‐3α, TP53 and CDK6. Let‐7g sequence (middle), wild‐type 3′UTR sequences (upper) and mutant 3′UTR sequences (lower) for let‐7g binding sites of (**A, B**) HIF‐3α, (**C**) TP53, and (**D**) CDK6. Noticeably, HIF‐3α has two let‐7g binding sties. Each data were from four independent repeated experiments. Data are presented as mean ± S.E.M., *P < 0.05, **P < 0.01, ***P < 0.001, comparing to the negative control miRNA mimic, two‐tailed *t*‐test.

Let‐7g is unlikely to directly bind to VEGF‐A since let‐7g increased VEGF‐A expression. We used the IPA software to indicate that let‐7g may directly bind to HIF‐3α and TP53, both of which are up‐stream regulators of VEGF‐A 16, 17, 18. The luciferase reporter assay confirmed that HIF‐3α harbours two let‐7g binding sites, and let‐7g could dose‐dependently reduce luciferase activity (Fig. 4A and B). Destruction either of the let‐7g binding sites in HIF‐3α 3′UTR aborted let‐7g's effect on luciferase activity. The luciferase assay also confirmed that let‐7g could bind to TP53 3′UTR leading to TP53 down‐regulation (Fig. 4C).

### Let‐7g enhances VEGF‐A alternative splicing through modulating CDK6 and SC35

**Figure 5:**
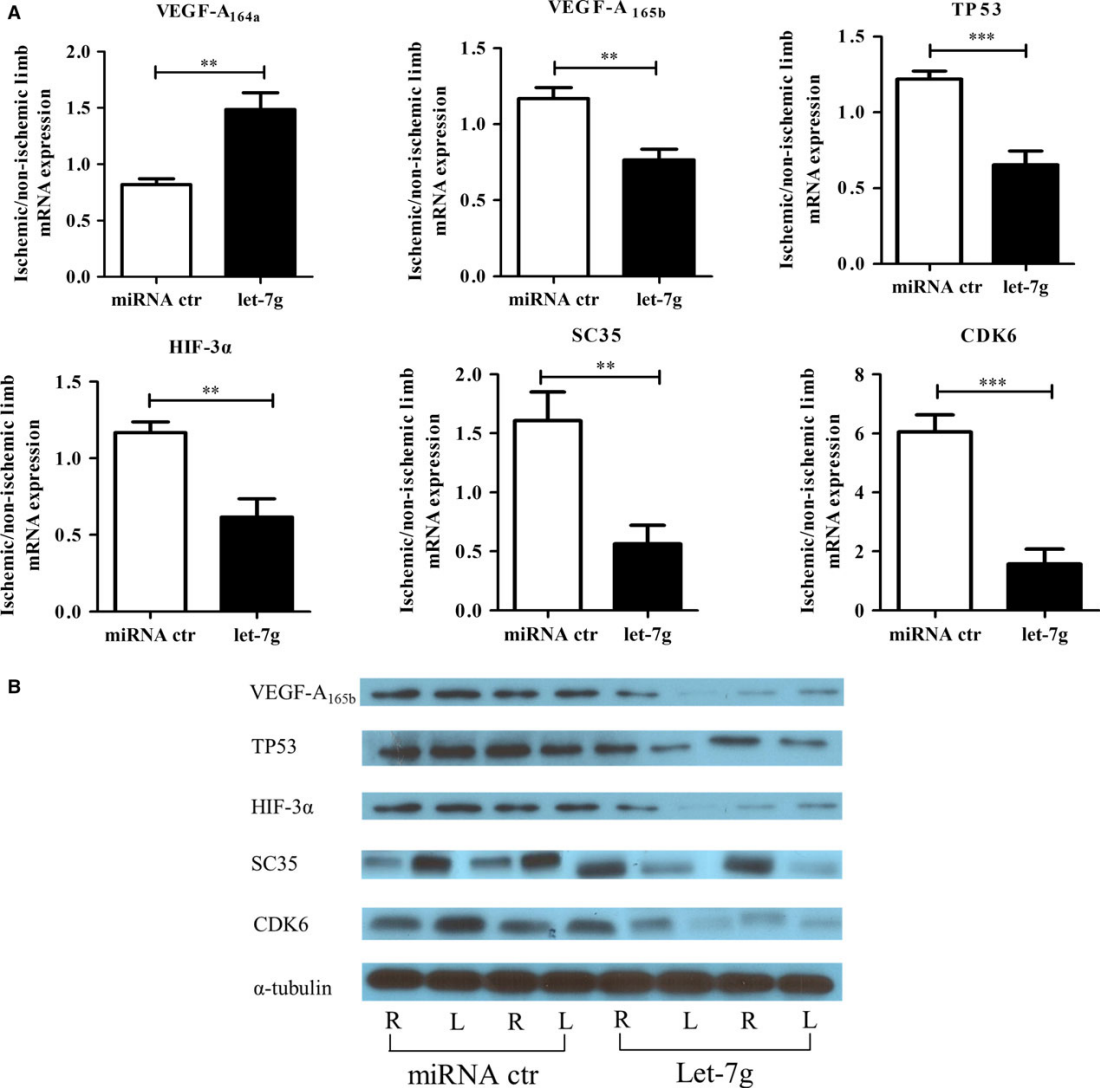
Let‐7g influenced the expression levels of VEGF‐A upstream genes and VEGF‐A splice isoforms. (**A**) The ratios of mRNA levels (ischaemic versus non‐ischaemic legs) of VEGF‐A upstream genes (HIF‐3α and TP53), VEGF‐A isoform determinants (CDK6 and SC35) and VEGF‐A two isoforms on day 21 after induced hindlimb ischaemia. (*n* = 6 for each group). (**B**) Western blot data show that the protein ratios are lower in the let‐7g‐treated than the miRNA control‐treated animals. Compared with the control group, the ratio in the let‐7g group is 0.34× for VEGF‐A_165b_, 0.70× for HIF‐3α, 0.68× for TP53, 0.59× for CDK6 and 0.20× for SC35 (*n* = 2 for each group). Data in the bar figures are presented as mean ± S.E.M., **P < 0.01, ***P < 0.001).

The splicing factor SC35 and the transciption factor E2F1 collaboratively control the alternative processing at exon 8 of VEGF‐A, which leads to VEGF‐A_164a_ (i.e. the rodent ortholog of human VEGF‐A_165a_) and VEGF‐A_165b_ isoforms 19. SC35 was found to be a transcription target of E2F1, and E2F1 increased the expression of SC35 in cellular models 19, 20. Using IPA, let‐7g was predicted to directly suppress CDK6, which inhibited the release of E2F1 and subsequently down‐regulated SC35 expression 21, 22. Luciferase reporter assay confirmed that let‐7g could directly knock down CDK6 (Fig. 4D). The *in vivo* data showed that the mRNA ratio of HIF‐3α, TP53, CDK6 and SC35 in the gastrocnemius muscle of ischaemic/non‐ischaemic hindlimb were lower in the let‐7g treated group than placebo group (*P* < 0.005) (Fig. 5A). Let‐7g treatment increased the ratio of pro‐angiogenic VEGF‐A_164a_ in ischaemic/non‐ischaemic hindlimb, but decreased the ratio of anti‐angiogenic VEGF‐A_165b_ levels (Fig. 5A). Western blot analysis showed similar findings as mRNA data. The ratios of protein amounts of VEGF‐A_165b_, HIF‐3α, TP53, CDK6 and SC35 were lowered by let‐7g when compared to the ratios in the placebo‐treated animals (Fig. 5B). Using the ratio from placebo‐treated animals as the reference, the relative ratios for let‐7g‐treated animals were 0.34 for VEGF‐A_165b_, 0.70 for HIF‐3α, 0.68 for TP53, 0.59 for CDK6 and 0.20 for SC35. Because no commercially available antibody for VEGF‐A_164a_, there was no VEGF‐A_164a_ protein data in Fig. 5B.

### Down‐regulation of CDK6 affects VEGF‐A alternative splicing machinery

**Figure 6:**
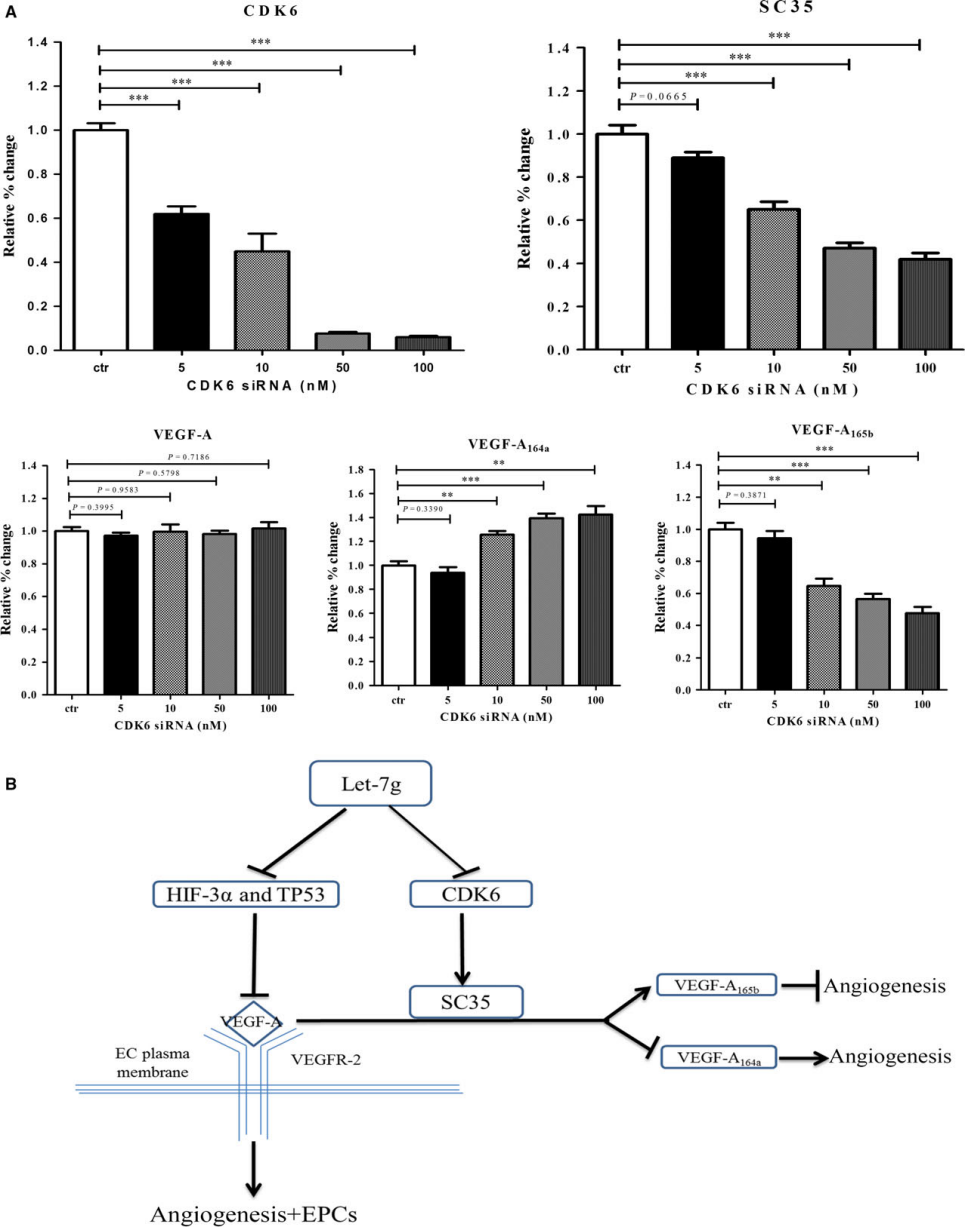
Down‐regulation of CDK6 affects VEGF‐A alternative splicing machinery. (**A**) Knockdown of CDK6 by siRNA suppresses CDK6 and SC35. Although the mRNA levels of total VEGF‐A were not changed, the pro‐angiogenic isoform VEGF‐A_164a_ increased dose‐dependently by CDK6 knockdown while an opposite pattern was found for anti‐angiogenic isoform VEGF‐A_165b_. Data are presented as mean ± S.E.M., **P < 0.01, ***P < 0.001, (**B**) Hypothetic schema shows multiple regulatory pathways of let‐7g on angiogenesis in ischaemic hindlimb model.

Our luciferase reporter assay and *in vivo* protein/mRNA expressions suggested that let‐7g could directly knock down CDK6 that is an up‐stream regulator of splicing factor SC35 21, 22. To confirm that down‐regulation of CDK6 can promote pro‐angiogenic isoform VEGF‐A_164a_ and inhibit anti‐angiogenic isoform VEGF‐A_165b_, C2C12 cells were transfected with different doses of CDK6 siRNA. As shown in Fig. 6A, the expressions of CDK6 and SC35 were dose‐dependently inhibited by CDK6 siRNA. Although the mRNA levels of total VEGF‐A were not changed, the pro‐angiogenic isoform VEGF‐A_164a_ increased dose‐dependently by CDK6 knockdown while an opposite pattern was found for anti‐angiogenic isoform VEGF‐A_165b_.

### Hypoxic effect on muscle cells

When C2C12 cells were subjected to either hypoxia or OGD, the expressions of all tested molecules (VEGF‐A, VEGFR‐2, VEGF‐A_164a_, VEGF‐A_165b_, TP53, HIF‐3α, CDK6 and SC35) were increased (Fig. S1).

## Discussion

Therapeutic angiogenesis has been pursued as a potential treatment for ischaemic disorders like PAD. Using cellular studies, we and others have demonstrated that let‐7g can induce angiogenesis 10, 11. The present study showed that let‐7g increased VEGF‐A expression by regulating HIF‐3α and TP53. MiRNA let‐7g also enhanced the alternative splicing of VEGF‐A towards the pro‐angiogenic isoform VEGF‐A_164a_ *via* targeting in the 3′UTR of CDK6 that further affects splicing factor SC35. Furthermore, let‐7g treatment increased bone marrow derived and circulating EPCs. These effects are summarized as a hypothetic schema in Fig. 6B.

Several regulatory pathways can contribuite to the pro‐angiogenic effects of let‐7g. Improved local perfusion through neo‐vasculization as indicated by the increase in capillary density could be attributed to up‐regulation of pro‐angiogenic genes (VEGF‐A and VEGFR‐2). The present study revealed dual regulatory mechanisms for let‐7g's modulation on the VEGF‐A signalling. First, let‐7g increased the expression of total VEGF‐A by direct repression of HIF‐3α and TP53. Secondly, leg‐7g suppressed the expression of alternative splicing factor SC35 by inhibiting its up‐stream regulator CDK6. Down‐regulation of CDK6 and SC35 further resulted in an increase in pro‐angiogenic isoform VEGF‐A_164a_ and a decrease in anti‐angogenic isoform VEGF‐A_165b_. On the other hand, both the bone marrow derived and circulating EPCs were significantly increased by let‐7g treatment. VEGF‐A can release EPCs from the bone marrow into peripheral circulation 23, 24, and circulating EPCs can incorporate into capillaries and interstitial arteries for neo‐vasculization 25. The multiple mechanisms indicate the potential of let‐7g in treating ischaemic diseases.

VEGF‐A is the major factor for angiogenesis and neo‐vasculization and it is up‐regulated as an adaptive response to ischaemic and hypoxic injury. Several hypoxia regulated/responsive elements located in the 5′ and 3′ UTR of the VEGF‐A gene are involved in the fine‐tuning of VEGF‐A expression 26. HIF‐1α and HIF‐2α are transcription factors that activate VEGF‐A expression, whereas HIF‐3α exerts a negative regulation of the angiogenic response by competing with HIF‐1α and HIF‐2α binding sites 16. In the present study, we demonstrated that let‐7g could bind two binding sites in the HIF‐3α 3′UTR to silence HIF‐3α expression. It might explain why let‐7g treatment led to increase VEGF‐A. In addition, we found let‐7g could also repress TP53 expression *via* annealing to its 3′UTR. TP53 has been shown to inhibit VEGF‐A promoter activity to down‐regulate VEGF‐A expression 17, 18. Furthermore, TP53 could also up‐regulate thrombospondin‐1 (also known as THBS1) that is an angiogenesis inhibitor 27. Our previous study has also shown that let‐7g could directly bind to THBS1 3′UTR and knock down its expression 10. A recent study showed that several let‐7 family members have hypoxia regulated/responsive elements in their promoters, and HIF‐1α‐induced let‐7 could directly suppress AGO1 11. AGO1 is involved in miRNA‐mediated silencing complex (miRISC) that causes gene silencing. VEGF‐A is translationally suppressed by AGO1‐miRISC under normoxia and let‐7 can increase VEGF‐A by repressing AGO1‐miRISC leading to VEGF‐A RNA release 11. Accordingly, let‐7g has multiple routes to increase VEGF‐A.

By alternative splicing of eight exons within the VEGF‐A gene, at least 14 different isoforms have been reported and they can be grouped into two families: the proangiogenic (denoted as VEGF‐Axxxa family) and the antiangiogenic (denoted as VEGF‐Axxxb) family), xxx where refers to the number of amino acids 28. The opposite effects of VEGF‐A splice isoforms on angiogenesis might be an important factor for VEGF‐A therapy. Because of the critical role of VEGF‐A in ischaemic diseases, there were several clinical trials of VEGF‐A gene therapy for PAD. However, limited success was achieved in clinical outcome 29. Interestingly, it has been known that PAD patients had elevated VEGF‐A levels but poor neo‐vasculization in legs 12. Further studies showed that PAD patients had elevated serum levels of the anti‐angiogenic isoform VEGF‐A_165b_ but reduced serum levels of the pro‐angiogenic isoform VEGF‐A_165a_ (the human ortholog of murine VEGF‐A_164a_) 12. VEGF‐A_165b_ competes with VEGF‐A_165a_/ VEGF‐A_164a_ for the binding to VEGFR‐2 28. In the present study, let‐7g treatment increased VEGF‐A_164a_ levels but decreased VEGF‐A_165b_ levels in murine ischaemic limb model. The ratio of VEGF‐A_165b_ and VEGF‐A_164a_ can be determined by SC35, which is a pre‐mRNA splicing factor for alternative processing at VEGF‐A exon 8 19. The SC35 protein was reported to increase the VEGF‐A_165b_/VEGF‐A ratio and to decrease tumour neovascularization in immune‐deficient nude mice 19. Our data demonstrated that let‐7g down‐regulated the CDK6/SC35/VEGF‐A_165b_ cascade and up‐regulated the expression of total VEGF‐A as well as the VEGF‐A_164a_ isoform. These regulatory machinery enforces the potential application of let‐7g in treating PAD.

To sum up, the present study showed that let‐7g intramuscular injection might be a potential therapeutic strategy in PAD. The proangiogenic effects of let‐7g *via* multiple mechanisms imply that let‐7g may possess a therapeutic potential for neovascularization in ischaemic diseases. Further studies are warranted to test the therapeutic effects for its clinical utility.

## Conflicts of interest

The authors confirm that there are no conflicts of interest.

## Supporting information


**Fig. S1.** Hypoxic effect on gene expression levels in muscle cells.
