# Supplementary material for: MicroRNA let‐7g possesses a therapeutic potential for peripheral artery disease
Source: J Cell Mol Med. 2016 Oct 3;21(3):519–29. doi: 10.1111/jcmm.12997 (PMC5323674; doi:10.1111/jcmm.12997)
Supplement: Supplementary file 1 — Fig. S1. Hypoxic effect on gene expression levels in muscle cells. [file JCMM-21-519-s001.docx]

**Supplementary Fig 1**
